# Effect of Side Substituent on Comb-like Polysiloxane Membrane Pervaporation Properties During Recovery of Alcohols C2-C4 from Water

**DOI:** 10.3390/polym16243530

**Published:** 2024-12-18

**Authors:** Evgenia Grushevenko, Islam Chechenov, Tatyana Rokhmanka, Tatiana Anokhina, Stepan Bazhenov, Ilya Borisov

**Affiliations:** 1 A.V. Topchiev Institute of Petrochemical Synthesis, Russian Academy of Sciences, Leninsky Prospect 29, 119991 Moscow, Russia; evgrushevenko@ips.ac.ru (E.G.); chechenov@ips.ac.ru (I.C.); rokhmankatn@ips.ac.ru (T.R.); tsanokhina@ips.ac.ru (T.A.); sbazhenov@ips.ac.ru (S.B.); 2 Federal Research Center Kazan Scientific Center of Russian Academy of Sciences, Lobachevskogo St. 2/31, 420111 Kazan, Tatarstan, Russia

**Keywords:** comb-like polysiloxane, pervaporation, alcohol recovery, water treatment, polymeric membrane

## Abstract

The pervaporation properties of membranes based on comb-like polysiloxanes when C_2_-C_4_ alcohols are removed from water were studied for the first time. It was established that membranes based on comb-like polysiloxanes with linear aliphatic and organosilicon substituents have increased permeability selectivity for C_3+_ alcohols. The obtained results were interpreted from the point of view of the solubility of the components of the separated mixture in polysiloxanes. It was shown that membranes based on polysiloxanes with linear substituents have increased butanol/water permeability selectivity (2.5–3.7). The achieved selectivity values correspond to the level of highly selective zeolite membranes, which allows for a reduction in energy consumption for the pervaporation removal of butanol by more than two times.

## 1. Introduction

The wastewater treatment of various chemical industries is an important environmental issue. One of the types of wastewater pollutants are oxygenates (ethers and lower alcohols). These substances are present in the effluents of GTL processes, namely petrochemical production (for example, effluents of the isomerization process).

Alcohols are an important intermediate, final, and by-product of industrial organic synthesis. For instance, the effluents of GTL processes contain a great number of lower alcohols, C_1_-C_5_ (22.8 g/L) [1]. These substances are not concentrated as a separate value-added product but are processed together with the total water effluent in the flotation and floaculation unit to remove suspended solids, followed by processing in a bioreactor, in which organic matter is disposed of to the level that allows wastewater to be discharged into reservoirs [2].

In the case of the targeted production of alcohols, they often need to be concentrated from aqueous media. According to various data, up to 50% of C_2_-C_5_ alcohols are formed in the aqueous phase during the synthesis of alcohols from CO and hydrogen, while the content of C_2_-C_4_ alcohols reaches 60–70% [3,4,5]. To obtain a marketable product, the alcohols obtained must be concentrated.

Pervaporation, or evaporation through a membrane, is one of the most promising methods of recovery of bio-alcohols from water [6]. It has advantages such as the absence of reagents, recovery of products at low temperatures, and modularity of the separation unit [7]. The main advantage of pervaporation over biopurification is the possibility of recovery and further use of alcohols.

There is a large number of works on the recovery of oxygen-containing organic compounds from aqueous media by pervaporation [6,7,8,9,10,11,12,13,14,15,16,17]. However, the number of membrane materials used for the recovery of oxygenates from aqueous media is small, comprising hydrophobic silicalites [8,17], silicone rubbers (for example, PDMS, POMS) [9,10,11], and highly permeable polymer glasses (for example, PTMSP, PIM-1) [12,13].

First of all, polydimethylsiloxane (PDMS) is used as a material of pervaporation membranes due to its high permeability, stable transport properties, and chemical and thermal resistance. The high flexibility of the PDMS chain, as well as weak intermolecular interactions, provide increased values of the free volume fraction and segmental mobility of the polymer, which determines its high gas and liquid permeability. The transport of gasses and vapors through nonporous membranes is determined by the “solution-diffusion” mechanism. This polymer has a record low diffusion selectivity [18]. Together with high solubility selectivity, it provides the increased selectivity of the permeability of PDMS membranes for components with increased solubility coefficients, and the chemical cross-linking of the polymer ensures the stability of the membranes in the media being separated. Polysiloxane membranes are widely used to purify liquid and gas streams from volatile organic compounds (VOCs). PDMS membranes have high permeability and stable transport properties, but their selectivity is insufficient to effectively solve many urgent separation problems. In this regard, the development of methods to increase the selectivity of polysiloxane membranes is a major challenge in this field of membrane science and technology.

Membranes based on polysiloxanes represent a subject of significant scientific and practical importance, particularly in the context of hydrocarbon capture and hydrophobic pervaporation processes [4], owing to their exceptional permeability and thermal stability [19]. Moreover, their cross-linked structure confers them with remarkable chemical stability in a wide range of solvents [20,21]. The separation capabilities of polydimethylsiloxane (PDMS) and polyoctylmethylsiloxane (POMS) membranes have been extensively explored [22,23,24,25]. Researchers have explored the potential of chemically modifying polysiloxane chains to enhance membrane separation performance. Of particular interest is the process of hydrophobizing polysiloxanes, which involves introducing alkyl side substituents with lengths ranging from 7 to 10 atoms into the polysiloxane backbone. This modification significantly improves the separation efficiency, as demonstrated by an increase in the selectivity factor for the separation of 1% weight fraction of methyl-tret-butyl ether from water, ranging from 111 for the methyl group to 161, 169, and 180, respectively. The incorporation of 10% diacetate groups into the polysiloxane backbone resulted in a 15% enhancement of the phenol flux from an aqueous solution compared to polydimethylsiloxane (PDMS), resulting in an increase in the phenol–water partition coefficient to 21.5. When separating a mixture of methyl isobutyl ketone and water, it was demonstrated that the addition of long alkyl substituents, such as octyl and tridecyl, significantly enhanced the separation efficiency, with partition coefficients increasing from 705 in PDMS to 1030 for octyl and 1200 for tridecyl.

Membranes based on polysiloxanes are of great scientific and practical interest, in particular for hydrocarbon capture and hydrophobic pervaporation [26], due to their high permeability and thermal stability [27]. In addition, they are chemically stable in a large number of solvents due to their cross-linked structure [28,29]. The separation ability of polydimethylsiloxane (PDMS) and polyoctylmethylsiloxane (POMS) membranes has been extensively studied [30,31,32,33]. Chemical modification of the polysiloxane chain can improve the separation characteristics of membranes. In particular, the hydrophobization of polysiloxanes is of great interest to researchers. Thus, the introduction of alkyl side substituents of lengths 7, 8, and 10 into the polysiloxane chain allowed for an increase in the separation factor of 1 wt.% methyl-tretbutyl ether in water from 111 (for the methyl group) to 161, 169, and 180, respectively [34]. The introduction of 10% diacetate groups into the polysiloxane chain allowed for an increase in the phenol flow from an aqueous solution by 15% compared with polydimethylsiloxane, which led to an increase in the phenol/water separation factor to 21.5 [35]. When separating a mixture of methylisobutyl ketone–water, it was shown that the introduction of long alkyl substituents (octyl, tridecyl) led to a significant increase in separation efficiency; the separation factor increased from 705 for PDMS to 1030 (octyl) and 1200 (tridecyl) [35].

Numerous studies have shown that polysiloxanes containing long linear aliphatic substituents have increased selectivity in the recovery of VOCs from water compared with other polysiloxanes [26]. The vast majority of works on the study of gas transport and pervaporation properties of polymers of this type are devoted to polyoctylmethylsiloxane (POMS) [10]. It was demonstrated in [32] that the introduction of a long hydrocarbon substituent C_8_ into the side chain of polysiloxane leads to an increase in the hydrophobicity of the membrane material (water flow for a PDMS membrane is 117 g·m^−2^·h^−1^, for a POMS membrane—85 g·m^−2^·h^−1^). Due to this, the selectivity of the recovery of non-polar aromatic substances in relation to water increases. However, it is unclear from the open sources what caused the choice of this polymer, since work on the directional design of comb-like polysiloxanes for the recovery of VOCs from water has not yet been carried out. This is largely due to the complexity of the synthesis of the membrane materials of this type. There are several methods for the synthesis of comb-like polysiloxanes and membranes on their basis [26]. All of them are multi-stage and require the synthesis of expensive monomers, the recovery of the polymer from the reaction mass, and the use of various catalysts for the synthesis of the polymer and its cross-linking [26]. The creation of an effective and simple method for the synthesis and cross-linking of polysiloxanes with different chemical structures and side chain length made it possible to synthesize a representative number of comb-like polysiloxanes and conduct systematic studies of the fundamental relationship between the structure and transport properties of this important group of membrane materials for hydrocarbons [36]. Thus, the successful solution of the problem of developing new and simple methods for the molecular design of polysiloxanes opens up opportunities for creating materials with increased selectivity during the pervaporation recovery of VOCs from water.

This work is aimed at studying the influence of the geometry of the side substituent of polymethylsiloxanes on their pervaporation properties during the recovery of lower alcohols from aqueous media. The novelty of the work lies in the establishment of a fundamental relationship between the chemical structure of side chains and the transport properties of membrane materials based on comb-shaped polysiloxanes for obtaining highly selective membranes in the process of the pervaporation separation of alcohols from water.

## 2. Materials and Methods

### 2.1. Materials

For the synthesis of polymethylorganosiloxanes, polymethylhydrosiloxane (PMHS) was used with a number average molecular weight, Mn, of 1700–3200 g/mol (Sigma-Aldrich, St. Louis, MO, USA), Karstedt’s catalyst (platinum complex of 1,3-divinyl-1,1,3,3-tetramethyldisiloxane in xylene, Sigma-Aldrich, St. Louis, MO, USA), 1-hexene (97%, Sigma-Aldrich, St. Louis, MO, USA), 1-heptene (97%, Sigma-Aldrich, St. Louis, MO, USA), 1-octene (98%, Sigma-Aldrich, St. Louis, MO, USA), 1-decene (94%, Sigma-Aldrich, St. Louis, MO, USA), 1-dodecene (95%, Sigma-Aldrich, St. Louis, MO, USA), 1-tetradecene (92%, Sigma-Aldrich, St. Louis, MO, USA), 3,3-dimethylbutene-1 (95%, ABCR, Karlsruhe, Germany), 4,4-dimethylpentene-1 (99%, Sigma-Aldrich, St. Louis, MO, USA), vinyltrimethylsilane (97%, ABCR, Karlsruhe, Germany), allyltrimethylsilane (98%, ABCR, Karlsruhe, Germany), 1,7-octadiene (98%, Sigma-Aldrich, St. Louis, MO, USA), n-hexane (99%, Chimmed, Podolsk, Russia) without further purification. To obtain polydimethylsiloxane, a vinyl-terminated polydimethylsiloxane (PDMS) was used at Mn 25,000 g/mol (Sigma-Aldrich, St. Louis, MO, USA), polymethylhydrosiloxane (PMHS) at Mn 1700–3200 g/mol (Sigma-Aldrich, St. Louis, MO, USA), Karstedt catalyst (platinum complex of 1,3-divinyl-1,1,3,3-tetramethyldisiloxane in xylene, Sigma-Aldrich, St. Louis, MO, USA), and toluene (99.8%, Chimmed, Podolsk, Russia) without further purification.

Ethanol (96.3%, Ferein, Moscow, Russia), propanol-1 (Chimmed, Podolsk, Russia), n-butanol (Chimmed, Podolsk, Russia), and distilled water were used to study the pervaporation membrane properties.

### 2.2. Synthesis and Production of Membranes

**Figure 1:**
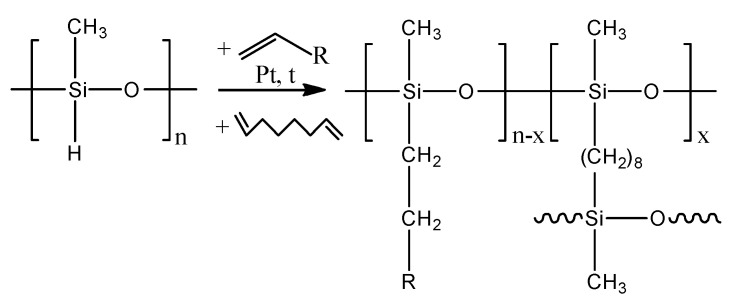
The scheme of the reaction for obtaining polymethylsiloxanes substituted by a side group. n—polymerization degree of initial PMHS, x—monomer units entering into a cross-linking reaction.

A molding solution and polymethylsiloxane membranes with various side groups were obtained using a recently proposed method [37]. This method consists of the in situ modification and cross-linking of polymethylhydrosiloxane (PMHS) with α-olefin (allyltrimethylsilane, 3,3-dimethylbutene-1, 1-hesene, 1-heptene (1-octene, 1-decene, 1-dodecene, 1-tetradecene) and diene hydrocarbon (1,7-octadiene) in the presence of a Karstedt catalyst by a hydrosilylation reaction. The reaction scheme is shown in Figure 1.

According to the method, the reaction was carried out in an n-hexane medium. For this purpose, a 3 wt.% solution of PMHS was pre-prepared in it. The reaction was carried out for 3 h while the solution was continuously stirred at 60 °C under a reflux condenser. The membranes were obtained by watering the reaction mixture on a stainless-steel mesh (cell size 40 μm) fixed on a Teflon surface, followed by drying to a constant weight at 60 °C for 24 h. The thickness of the films varied in the range of 40–50 μm. A polydimethylsiloxane (PDMS) membrane was used as an object of comparison: vinyl-terminated PDMS was cross-linked with PMHS by a hydrosilylation reaction in the presence of a Karstedt catalyst. The ratio of PDMS:PMHS:catalyst was 10:1:0.01. The films on the mesh were obtained by the method described above. The conventional symbols of the membranes obtained are shown in Table 1.

### 2.3. Vacuum Pervaporation

**Figure 2:**
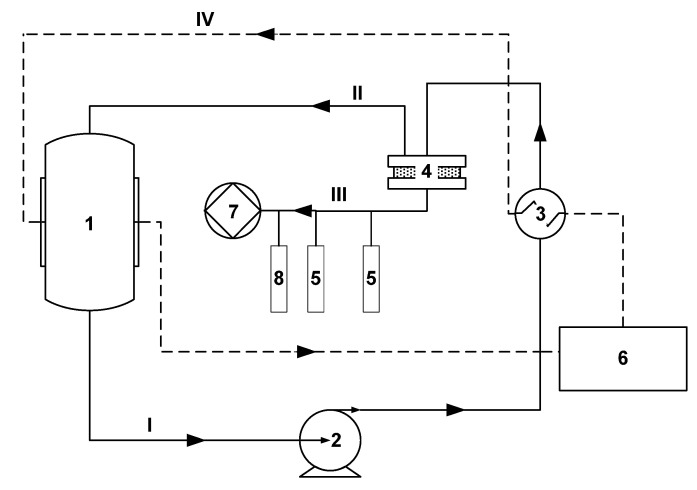
Layout of the vacuum pervaporation installation [19].

The pervaporation experiments were carried out on the installation shown in Figure 2 and described in detail in [19].

The initial feed mixture was poured into a 1 L container (1) and pumped in a circulating mode using an Ismatec gear pump (2). The volumetric flow rate of the feed mixture varied from 20 to 220 mL/min. The mixture heated in the heat exchanger (3) was fed into the membrane module (4). The effective membrane area was 13.85 cm^2^. Permeate vapors were condensed in glass traps placed in Dewar vessels with liquid nitrogen (−196 °C) (5). The continuous operation of the installation throughout the experiment was ensured by the presence of two parallel traps. To prevent permeate vapor from entering the vacuum pump, a safety trap was used (8). The temperature of the feed mixture was maintained with an accuracy of ±0.1 °C using a LOIP LT-100 liquid thermostat (6). To create the driving force of the mass transfer process in the submembrane space, a pressure of ~0.05 mbar was maintained with an Ebara PDV-250 vacuum pump (7).

The pervaporation was carried out at a temperature of the feed mixture of 30 (±0.1) °C. A solution of n-butanol, n-propanol, and ethanol in water with an organic component content of 1.0, 1.0, and 3.0 wt.%, respectively, was used as a feed mixture. The solution used was prepared from an organic solvent (purity grade–reagent grade) by the gravimetric method.

The concentration of the initial mixture and permeate was determined by gas chromatography on a Crystallux-4000M chromatograph equipped with a thermal conductivity detector.

The process of vacuum pervaporation was characterized using the following parameters: permeate flow, separation factor, and the index of pervaporation separation.

Total permeate flux (*J*, kg/m^2^ h) was calculated as

(1) $$J=\frac{m}{S\cdot t},$$

where *m* is the weight of the permeate (kg) penetrated through the membrane with the area *S* (m^2^) for a known period of time *t* (h).

The separation factor (α) was determined by the following formula:

(2) $$\alpha =\frac{y_{o}\cdot x_{w}}{y_{w}\cdot x_{o}},$$

where *x_o_* and *x_w_* are the mass fractions of the organic component and water in the separated mixture and *y_o_* and *y_w_* are the mass fractions of the organic component and water in the permeate.

It is worth noting that the parameters listed above depend on the experiment conditions, involving temperature, composition of the feed mixture, pressure in the submembrane space, and the type of membrane [20]. Therefore, it is preferable to compare the transport properties of membranes using such process parameters as permeability (P/l) and selectivity, which was determined from the ratio of the permeability of the *i*-th component to the permeability of water.

The permeability coefficient (*P*, Barrer) for component *i* was calculated according to the following equation:

(3) $$P=\frac{J_{i}l}{\left(P_{i}^{f}-P_{i}^{p}\right)},$$

where *J_i_* is the component *i* flow (cm^3^/(cm·m^2^·s)), $p_{i}^{f}$ and $p_{i}^{p}$ are the vapor pressure of component *i* in the initial mixture and permeate (cm hg), respectively, and *l* is the thickness of the membrane (m). To determine the vapor pressure of permeate and the initial mixture in the case of the binary mixtures of n-propanol–water and ethanol–water, activity coefficients were calculated using the Aspen Plus 8.6 software package using the NRTL (non-random two-liquid) model [21]. As it was shown in [22], in the case of the binary mixture of n-butanol–water, it is preferable to use the approximated 4-parametric Margules equation to calculate the activity coefficients.

(4) $$lgy_{b}=(1-x_{b}{)}^{2}\left[A+2\left(B-A-D\right)x_{b}+Dx_{b}^{2}\right],$$

(5) $$lgy_{w}=x_{b}[B+2\left(A-B-D\right)\left(1-x_{b}\right)+3D(1-x_{b}{)}^{2}\;,$$

### 2.4. Determination of Sorption and Solubility Coefficients

The solubility coefficients of butanol in the polymer were determined under isothermal conditions according to the method developed by us. Sorption was carried out in a solution of 1 wt.% of 1-butanol in water. A sample of polysiloxane with mass (m_pol_) and density (ρ_pol_) is placed in a closed space filled by half with a model mixture (V_0_). Next, the sample was thermostated (LOIP LT-108a thermostat, Saint-Petersburg, Russia) at 30 °C for 24 h. It was found out that sorption equilibrium is established during this time, since the concentration of butanol in the solution does not change in the subsequent time. The amount of the sorbed butanol substance was calculated as the difference between the initial amount of the butanol substance and the amount of butanol substance after 24 h of sorption in the solution. The amounts of the substance were calculated based on the mass concentrations of butanol in the solution, which were determined by gas chromatography. The concentration of butanol was determined by analyzing the equilibrium concentrations of the vapor phase in order to increase the accuracy of the analysis. The vapor phase analysis was performed using a headspace sampler (DRP-10, NPF Meta-Chrome LLC, Yoshkar-Ola, Russia), the main feature of which is the use of a thermostatically controlled gas syringe for sampling. This approach makes it possible to increase the measurement accuracy by increasing the area of peaks observed on the chromatogram. The initial mass concentration w_0_ in the solution and the mass concentration obtained after 24 h of sorption w_24_ were determined using a Crystallux-4000M gas chromatograph (NPF Meta-Chrome LLC, Yoshkar-Ola, Russia). The chromatographic analysis parameters were as follows: evaporator temperature—230 °C; column temperature—180 °C; and detector temperature—230 °C. The analyses were carried out using a 1 m long packed column filled with Porapak Q sorbent. The concentration obtained from the chromatograms w_24_ corresponds to the concentration of a sorbate in the solution after 24 h of contact with the polymer.

The amount of the substance (mol) of butanol was found by the following formula:

(6) $$n^{b}=\frac{w^{b}\cdot m_{s}}{M^{b}},$$

where *w^b^* and *M^b^* are a mass fraction and the molecular weight (g/mol) of butanol and *m_s_* is the mass of the solution (g).

The solubility coefficient (cm^3^/(cm^3^ cmHg) was calculated using the following formula:

(7) $$S_{i}=\frac{n_{s}\cdot R\cdot T}{V_{pol}\cdot p_{sorb}},$$

where *p_sorb_* and *T* are the sorbate vapor pressure (cmHg) and temperature (K).

Polymer volume (cm^3^), as the ratio of polymer mass and density:

(8) $$V_{pol}=m_{pol}/\rho_{pol},$$

where *m_pol_* (g) and *ρ_pol_* (g/cm^3^) are the polymer mass and density.

The amount of the substance sorbed in the polymer (mol) was determined as

(9) $$n_{s}=n_{0}-n_{24},$$

The solubility of water in the polymer was determined gravimetrically. The polymer was weighed in dry air and saturated water vapor at 30 °C on a Sartorius ENTRIS124-1S scale. The amount of the substance of the sorbed water was determined by the following formula:

(10) $$n^{w}=\frac{{m_{pol}^{24}-m}_{pol}^{0}}{M^{w}},$$

where $m_{pol}^{0}$ and $m_{pol}^{24}$ are the mass of the polymer in the dry state and in water vapor (g) and *M^w^* is the molecular weight of water (g/mol).

### 2.5. Scanning Electron Microscopy (SEM)

SEM was used to characterize the structure and morphology of the membranes. SEM was carried out on a Thermo Fisher Phenom XL G2 Desktop SEM (Thermo Scientific, Waltham, MA, USA). Cross-sections of the membranes were obtained by fracturing in liquid nitrogen after a preliminary impregnation of the specimens in isopropanol. A thin (5–10 nm) gold layer was deposited on the prepared samples in a vacuum chamber (~0.01 mbar) using a desktop magnetron sputter “Cressington 108 auto Sputter Coater” (Cressington Scientific Instruments Ltd., Watford, UK). The accelerating voltage during image acquisition was 15 kV.

## 3. Results and Dicussion

### 3.1. Synthesis of Membranes from Comb-like Polysiloxane

The proposed one-step method for the synthesis of film and composite membranes based on comb-like polysiloxanes consists of in situ modification and cross-linking, excluding the stages of the separation and purification of the polymer between the stages of modification and cross-linking (Figure 1). A distinctive feature of the proposed method is that the modification and cross-linking reaction is carried out in the same reaction medium in the presence of a single Pt-containing catalyst (Karstedt catalyst). An important simplification of this approach to the synthesis of polysiloxane membranes is the use of cheap and commercially available starting compounds, such as polymethylhydrosiloxane (a product of organosilicon production), α-olefins, and dienes (products of basic organic synthesis). The main advantages of the new approach in comparison with traditional methods for obtaining comb-like polysiloxanes are as follows:

- The use of the reaction mixture as a molding solution;
- The use of a single catalyst for the modification and cross-linking of the membrane material;
- The absence of stages of polymer recovery and purification (low solvent consumption);
- The simplicity of the modification of the material chemical structure (variability in the use of modifying agents with terminal double bonds);
- Little time spent on membrane production.

**Figure 3:**
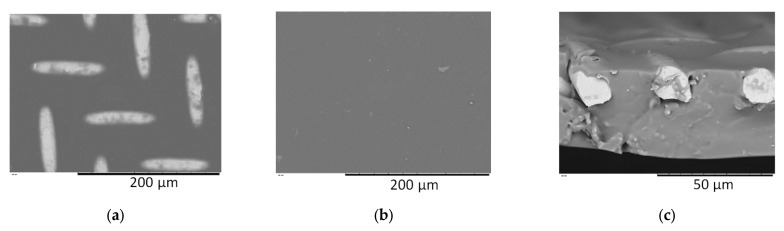
SEM images of a polysiloxane membrane on a stainless-steel mesh: (**a**) top view, (**b**) bottom view, (**c**) cross-section.

Using a new method, a series of 10 membranes based on comb-like polysiloxanes with linear and branched structures, as well as silicon-containing side substituents, were synthesized and studied in the process of pervaporation (Figure 1). Typical SEM images of obtained membranes are represented in Figure 3.

### 3.2. Transport Properties of the Obtained Membranes Based on Comb-like Polysiloxane

**Figure 4:**
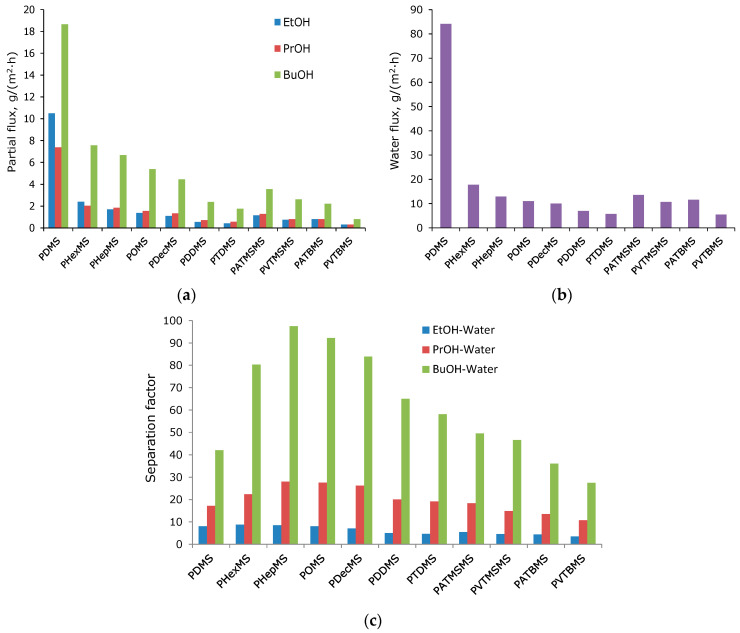
(**a**) The dependence of the component fluxes on the type of side substituent (BuOH—1-butanol; PrOH—1-propanol; EtOH—ethanol). (**b**) the dependence of the water flux on the type of side substituent. (**c**) The dependence of the alcohol–water separation factor on the type of side substituent (BuOH—1-butanol; PrOH—1-propanol; EtOH—ethanol; W—water).

The transport properties of the obtained membranes were studied during the pervaporation separation of a model mixture of n-butanol, n-propanol, and ethanol in water with an organic component content of 1.0, 1.0, and 3.0 wt.%. The experimental results are shown in Figure 4 and Table 2.

In Figure 4a,b, there is a tendency to decrease the partial flows of components with an increase in the size of the side substituent. In the case of polysiloxanes with linear side chain substituents, the flow decreases monotonously with increasing side chain length. For polysiloxanes with branched substituents, the flow increases with increasing length of the flexible aliphatic junction between the bulk group (tertbutyl or trimethylsilyl) and the main chain of two to three methylene fragments. This behavior is associated with a decrease in conformational rotation when the bulk group is removed from the main chain. Moreover, the fluxes for polysiloxanes containing a trimethylsilyl fragment in the side chain are higher than for those containing a tertbutyl fragment, which may be a consequence of the high mobility of the trimethylsilyl group [23,24]. The PDMS membrane (the most permeable in the series being studied) is characterized by a high water flux value, which exceeds the value of the 1-butanol flux by more than 2.5 times, and in the case of propanol and ethanol, by 5 and 10 times. In the case of comb-like polysiloxanes, the ratio of water and alcohol fluxes decreases sharply compared to PDMS, which leads to a proportional increase in the alcohol–water separation factor (Figure 4c). This can be explained by an increase in the hydrophobicity of the material with the introduction of large hydrocarbon side substituents [32].

Thus, PDMS is characterized by relatively low values of the separation factor (BuOH-W—42; PrOH-W—17; EtOH-W—8.5). For linear polyalkylsiloxanes, the C3-C4 alcohol separation factors are significantly higher (BuOH-W—58–97; PrOH-W—19–28 (Table 2)). The EtOH-W separation factor varies slightly in the series of polysiloxanes being considered (7.0–8.5). The maximum value of separation factors was demonstrated by the PHepMS membrane (BuOH-W—97.5; PrOH-W—29; EtOH-W—8.5 (Table 2)). However, for branched polyalkylsiloxanes, the separation factors are significantly lower. For example, PVTBMS demonstrates minimum separation factor values, even lower than PDMS (BuOH-W—27.5; PrOH-W—10.7; EtOH-W—3.5). Flux and the separation factor are the parameters that depend on the magnitude of the driving force of the process and the thickness of the membrane. To explain these results, it is necessary to involve studies of the structure of the material, as well as the analysis of the results in terms of the permeability coefficients of polymers and their components (diffusion and solubility coefficients) [20].

**Figure 5:**
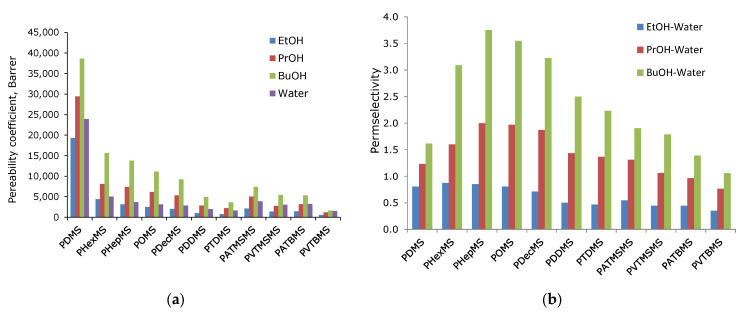
The dependence of the values of the permeability coefficients on the type of side substituent. (BuOH—1-butanol; PrOH—1-propanol; EtOH—ethanol; W—water (**a**). The dependence of the alcohol–water selectivity values on the type of side substituent. (BuOH—1-butanol; PrOH—1-propanol; EtOH—ethanol; W—water (**b**).

It can be seen from Figure 5 and Table 3 that the dependencies of the values of the permeability and selectivity coefficients correlate well with the data on the values of the fluxes of components and alcohol–water separation factors. Among the studied polysiloxanes, the most permeable membrane material is PDMS (P (BuOH) = 38,700 Barrer; P (PrOH) = 29,500 Barrer; P (EtOH) = 19,300 Barrer; P (H_2_O) 23,900 Barrer). As the length of the linear alkyl side substituent increases, the permeability decreases for all components (Figure 5a). However, a more favorable situation is observed for alcohols C_3_ and C_4_. For example, the selectivity for butanol is in the range of 2.5–3.7 for polyalkylsiloxanes with linear C_6_-C_12_ substituents, which corresponds to the highly selective zeolite membranes [25]. Such an increase in selectivity leads to a decrease in energy consumption for the pervaporation recovery of butanol by more than two times [25], which indicates the prospects of linear polyalkylsiloxanes for solving the problem of the pervaporation recovery of PrOH and BuOH from aqueous media. At the same time, the maximum selectivity of permeability for alcohols EtOH-W (0.85), PrOH-W (2.0), and BuOH-W (3.7) is demonstrated by the PHepMS membrane. A slight increase in ethanol selectivity is due to its high polarity and hydrophilicity. Indeed, PDMS derivatives have an EtOH/H_2_O selectivity either lower or close to one [25].

**Figure 6:**
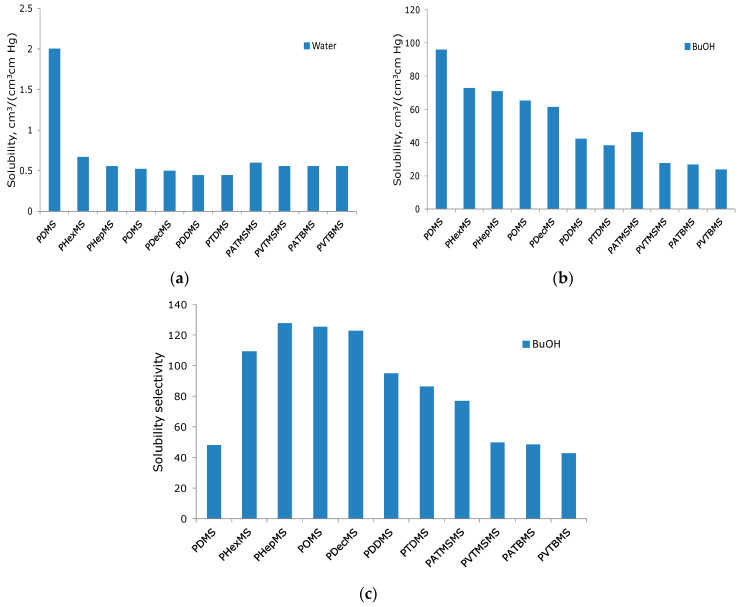
The dependence of the values of the water and butanol solubility on the type of side substituent (**a**,**b**). The dependence of the butanol–water solubility selectivity values on the type of side substituent (**c**).

The following question arises: what is the reason for the increased selectivity of linear polyalkylsiloxanes, and why is the selectivity of polysiloxanes with bulk substituents significantly lower than that of the former? The fact is that the selectivity of permeability is the product of the selectivity of diffusion and solubility. The preferred permeability of alcohols C3 and C4 compared with water in the polysiloxanes being studied is determined by significantly lower water solubility coefficients compared with butanol. As shown in Figure 5, the introduction of nonpolar side substituents reduces the water solubility coefficients in polysiloxanes compared to PDMS, whereas the solubility of butanol decreases not so significantly (Figure 6a,b). As a result, the selectivity of the solubility αs for PDMS and PHepMS differ 2.7 times (48 and 128, respectively) (Figure 6b). As can be seen from Figure 5, it is the selectivity of solubility that makes the main contribution to the selectivity of the alcohol/water permeability of polysiloxane membranes.

In each of the studied homologous series of polymers (with linear alkyl, trimethylsilyl, tertbutyl, and cyclohexyl substituents), the selectivity of solubility increases with an increase in the hydrocarbon fragment (Figure 6b). This is due to the fact that n-butanol is soluble in aliphatic hydrocarbons and the solubility of water in alkanes is close to zero. As was shown in our previous work [36], ordered structures are formed in polysiloxanes with linear substituents, which are nanoscale layers formed by parallel-oriented side chains. The proportion of such ordered areas in the polymer was estimated based on X-ray phase analysis data obtained at liquid nitrogen temperature. Such membrane materials are heterogeneous, and their transport properties are determined by the supramolecular structure of the comb-like polyalkylsiloxane. An increase in the proportion of the ordered domains in the polymer leads to a decrease in the selectivity of diffusion for small molecules and an increase in the selectivity of permeability for larger organic components.

Based on the results obtained, it is possible to assume the reason for the increased selectivity of alcohol/water polysiloxanes with linear substituents. As shown in [36], the selectivity of diffusion for small molecules of polymers with linear substituents is lower than for PDMS, and for comb-like polysiloxanes with bulk substituents, it is at the level of or significantly higher than PDMS. The low selectivity of water/alcohol diffusion is one of the factors ensuring the high selectivity of alcohol/water permeability of comb-like polysiloxanes with linear side substituents capable of forming a hexagonal packing of side chains. This can be explained by the fact that due to plasticization, the organic component is embedded in a hexagonal structure formed by side substituents, forming additional transport channels for the diffusion of molecules. Such a diffusion mechanism is unlikely for water, since its solubility in hydrocarbons is negligible. In its turn, such hexagonal packing is not implemented for side substituents of a nonlinear structure. Therefore, the selectivity of diffusion for polymers with bulk substituents is higher compared to linear polyalkylsiloxanes. As a result, the permeability selectivity of polymers with bulk substituents is lower than with linear ones. In addition, for polymers, the tertbutyl side groups are even lower than for PDMS (Figure 6).

## 4. Conclusions

The pervaporation properties of membranes based on comb-like polysiloxanes were studied during the separation of a four-component mixture of BuOH-PrOH-EtOH-H_2_O for the first time. It has been established that membranes based on comb-like polysiloxanes with linear aliphatic and organosilicon substituents have an increased selectivity of permeability to alcohols C_3+_, which can be more than twice as high as the selectivity of membranes based on polydimethylsiloxane due to an increase in the selectivity of alcohol/water solubility. The membrane based on polyheptylmethylsiloxane demonstrates the greatest selectivity (separation factors 97, 29, and 9 for 1-butanol, 1-propanol, and ethanol, respectively), which is consistent with the high solubility coefficients of alcohols in this material. It has been shown that membranes based on polysiloxanes with linear substituents have an increased butanol/water permeability selectivity (2.5–3.7) relative to membranes made of polymers with bulk substituents (1.0–1.9) due to the low selectivity of water/alcohol diffusion. The achieved selectivity values correspond to the level of highly selective zeolite membranes, which allows for a reduction in energy consumption for the pervaporation recovery of butanol by more than two times [25]. Thus, it can be argued that membranes based on polyalkylsiloxanes with linear C_6_-C_12_ substituents are promising for solving the problem of the pervaporation recovery of C_3+_ alcohols from aqueous media.

## Figures and Tables

**Table 1:** Conventional symbols of the samples of membranes under study.

Symbol of Membrane	Polymer
PHexMS	Polyhexylmethylsiloxane
PHepMS	Polyheptylmethylsiloxane
POMS	Polyoctylmethylsiloxane
PDecMS	Polydecylmethylsiloxane
PDDMS	Polydodecylmethylsiloxane
PTDMS	Polytetradecylmethylsiloxane
PVTBMS	Poly-3,3-dimethylbutylmethylsiloxane
PATBMS	Poly-4,4-dimethylpentene-1methylsiloxane
PVTMSMS	Poly-3,3-dimethylsilbutylmethylsiloxane
PATMSMS	Poly-4,4-dimethylsilpentylmethylsiloxane
PDMS	Polydimethylsiloxane

**Table 2:** Partial fluxes for 1-butanol, 1-propanol, ethanol, and water and alcohol–water separation factors for studied polysiloxane membranes.

Membrane	Partial Flux, g/(m^2^ h)				Separation Factor (X/Water)		
	BuOH	PrOH	EtOH	Water	BuOH	PrOH	EtOH
PDMS	18.7	7.4	10.5	84.1	42	17.2	8.1
PHexMS	7.6	2.0	2.4	17.8	80.4	22.4	8.7
PHepMS	6.7	1.8	1.7	12.9	97.5	28.0	8.5
POMS	5.4	1.6	1.4	11.0	92.3	27.5	8.1
PDecMS	4.4	1.4	1.1	10.0	83.9	26.2	7.1
PDDMS	2.4	0.7	0.5	6.9	65.0	20.1	5.0
PTDMS	1.8	0.6	0.4	5.7	58.1	19.1	4.7
PATMSMS	3.6	1.3	1.2	13. 6	49.5	18.3	5.5
PVTMSMS	2.6	0.8	0.8	10.7	46.5	14.9	4.5
PATBMS	2.2	0.8	0.8	11.6	36.1	13.5	4.4
PVTBMS	0.8	0.3	0.3	5.5	27.5	10.7	3.5

**Table 3:** Permeability coefficients for 1-butanol, 1-propanol, ethanol, and water and alcohol–water selectivity for studied polysiloxane membranes.

Membrane	Permeability Coefficient, Barrer				Separation Factor (X/Water)		
	BuOH	PrOH	EtOH	Water	BuOH	PrOH	EtOH
PDMS	38,700	29,450	19,330	23,930	1.6	1.2	0.8
PHexMS	15,600	8100	4420	5060	3.1	1.6	0.9
PHepMS	13,800	7360	3130	3680	3.8	2.0	0.9
POMS	11,140	6180	2530	3140	3.5	2.0	0.8
PDecMS	9200	5340	2025	2850	3.2	1.9	0.7
PDDMS	4930	2830	990	1970	2.5	1.4	0.5
PTDMS	3630	2220	760	1620	2.2	1.4	0.5
PATMSMS	7360	5060	2120	3870	1.9	1.3	0.5
PVTMSMS	5430	2760	1380	3040	1.8	1.1	0.5
PATBMS	5340	3190	1470	3250	1.4	1.0	0.4
PVTBMS	1660	1200	550	1570	1.1	0.8	0.4

## Data Availability

The original contributions presented in this study are included in the article. Further inquiries can be directed to the corresponding author.

## Abbreviations

y_i_, x_i_, w_i_	the component mass fraction [g ∙ g^−1^]
J_i_	component mass permeate flux [kg ∙ m^−2^ ∙ h^−1^]
l	membrane selective layer thickness [cm]
M_i_	molar mass of the component [kg ∙ mol^−1^]
P_i_	permeability coefficient [Barrer]
S_i_	solubility coefficient [cm^3^ ∙ cm^−3^ ∙ cm Hg^−1^)]
α_i/j_	separation factor alcohol/water [-]
n_i_	amount of substance [mol]
m_i_	mass of component [g]
V_i_	volume of component [cm^3^]
ρ_i_	density of component [g/m^3^]
BuOH	n-butanol
PrOH	n-propanol
EtOH	ethanol
W	water
